# Supplementary material for: Measuring Glycolytic Activity with Hyperpolarized [2H7, U-13C6] D-Glucose in the Naive Mouse Brain under Different Anesthetic Conditions
Source: Metabolites. 2021 Jun 23;11(7):413. doi: 10.3390/metabo11070413 (PMC8303162; doi:10.3390/metabo11070413)
Supplement: Supplementary file 1 [file metabolites-11-00413-s001.zip › metabolites-1213085 SI.pdf]

Supplementary Materials

Measuring glycolytic activity with hyperpolarized [$^{2}H_{7}$, U-$^{13}C_{6}$] D-glucose in the naive mouse brain under different anesthetic conditions

Emmanuelle Flatt[1†], Bernard Lanz[1†], Yves Pilloud[2], Andrea Capozzi[1], Mathilde H Lerche[3], Rolf Gruetter[1], Mor Mishkovsky[1*]

Table S1. Absolute metabolite concentrations from ^{1}H MRS.

	Isoflurane		Medetomidine + isoflurane		
**Metabolite**	**Mean**	**SD**	**Mean**	**SD**	***p*-value**
Cr	4.253	0.820	3.371	0.808	ns
Cr/PCr	0.897	0.283	0.559	0.176	0.041
Gln	3.425	1.430	2.998	0.973	ns
Glu	7.600	0.708	6.943	1.173	ns
Glx	11.025	1.890	9.951	1.497	ns
Ins	6.312	1.044	7.522	0.886	ns
Lac	4.070	0.554	2.416	0.929	0.001
NAA	7.541	0.427	6.832	0.790	ns
PCr	4.950	0.861	6.253	1.135	0.047
PE	2.682	0.299	2.3750	0.949	ns
Tau	9.337	0.750	9.247	0.615	ns

Absolute metabolite concentrations quantified by LCModel under the two anesthetic condition. Creatine (Cr), phosphoceratine (PCr), glutamine (Gln), glutamate (Glu), glutamine + glutamate(glx), myo-inositol (Ins), lactate (Lac), N-acetylaspartate (NAA), Phosphorylethanolamine (PE), Taurine (Tau). Mean ± standard deviation (SD), exact *p*-values of significantly different concentrations are reported (below 0.05) otherwise the difference considered not significant (ns).

Table 2. Baseline information on animals participating in the study (mean ± SD).

**Bolus**	**HP ^{13}C Glc**	**^{2}H Glc**	**HP ^{13}C Glc**	**^{2}H Glc**
# animals (a.u.)	8	5	6	6
Body weight (g)	26.7 ± 2.5	28.7± 3.6	27.6 ± 1.4	28.8± 2.1
Age (weeks)	17 ±2	18 ± 1	17 ± 4	18 ± 2
Glycaemia before MR scan (mM)	6.2 ± 2.8	9.7 ± 2.5	6.8 ± 1.5	7.4 ± 0.7
Glycaemia after MR scan (mM)	12.4 ±3.2	17.9± 4.4	12.7 ± 2.9	28.7 ± 2.3
^{13}C or ^{2}H Estimated fractional enrichment (%)	71 ± 10	93 ± 2	69 ± 6	95 ± 1
PO_2 before MR scan (mmHg)	88 ± 9	87 ± 6	82 ± 8	86 ± 13
PO_2 after MR scan (mmHg)	83 ± 22	75 ± 3	57 ± 10	56 ± 13
PCO_2 before MR scan	31 ± 5	38 ± 10	34 ± 7	35 ± 4

(mmHg)				
PCO_2 after MR scan (mmHg)	36 ± 4	45 ± 5	60 ± 4	64 ± 3
Blood lactate before MR scan (mM)	1.9 ± 0.7	2.6 ± 1.1	2.3 ± 1.1	2.0 ± 0.5
Blood lactate after MR scan (mM)	4.1 ± 1.0	3.6 ± 1.6	2.5 ± 0.7	1.1 ± 0.1

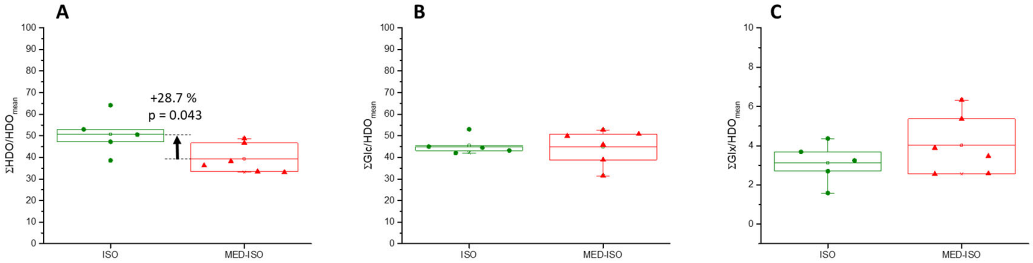

Figure S1. ^{2}H metabolite ratios after bolus of non-HP ^{2}H Glc in mice anesthetized by isoflurane (green) and a combination of medetomidine and isoflurane (red). Ratios were calculated based on the mean water signal before the bolus averaged over all animals (HDO_{mean}). (**A**) sum of ^{2}H water (ΣHDO) over HDO_{mean} (**B**) sum of glucose (ΣGlc) over HDO_{mean} (**C**) sum of glutamate + glutamine (Glx) over HDO_{mean}.

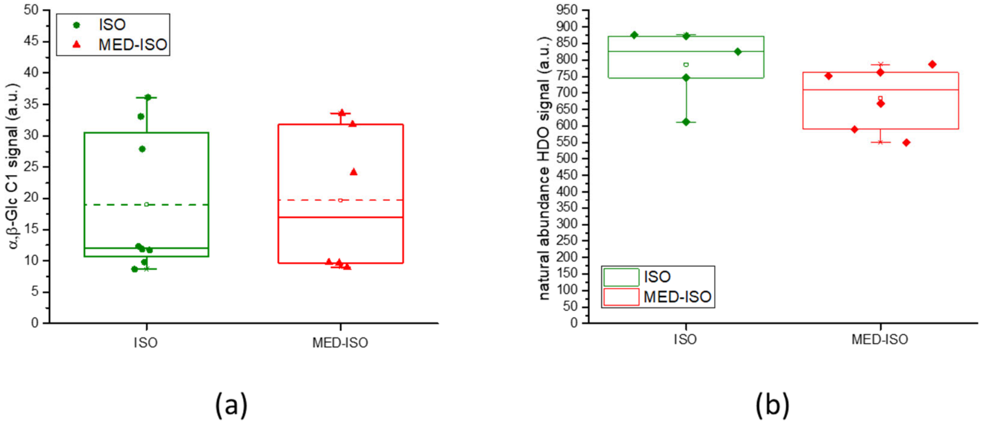

Figure S2. natural abundance HDO signal before injection. (**a**) signal intensity of the C-1 position of the HP ^{13}C glucose (α + β) from the sum over the entire array of spectra in animals anesthetized by isoflurane (green) and a combination of medetomidine with isoflurane (red). The averages were 550 ± 240 and 446 ± 226 a.u. (mean ± SD) respectively and were not significantly different (p = 0.42). The standard deviation of these values is mostly influenced by the in variability of the hyperpolarization levels of the tracer prior to the infusion due to the manual dissolution protocol. (**b**) HDO signal intensity before bolus of non-HP ^{2}H Glc in mice anesthetized by isoflurane (green) and a combination of medetomidine and isoflurane (red). The means values are 786 ± 110 and 685 ± 98 a.u. (mean ± SD) respectively. The means were not significantly different however a trend of higher HDO signal in the isoflurane group compared to MED-ISO group was observed (+15%, p = 0.14). This difference is likely related to the vasodilation properties of isoflurane.

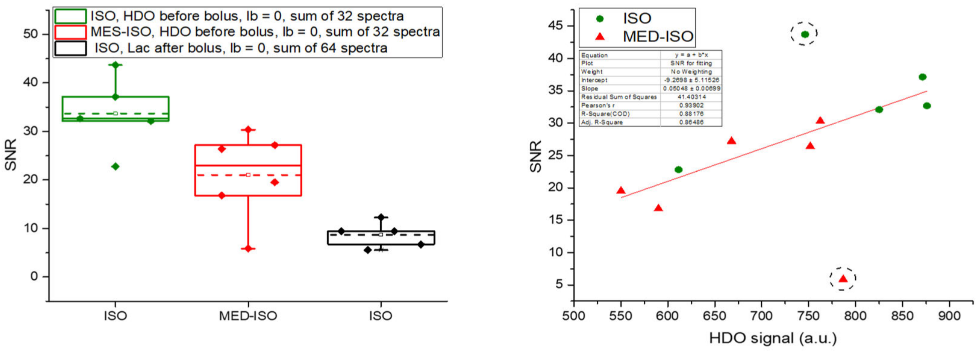

(a) (b)

Figure S3. Signal-to-noise ratio (SNR) analyses of ^{2}H MRS experiments (a) SNR comparison of HDO signal before the injection of glucose bolus without line broadening (LB = 0) on the sum of all 32 spectra. A significantly higher SNR was found in animals in the ISO group (green) than in MED-ISO group (red) with mean and standard deviation values of 33.7 ± 7.7, and 21.0 ± 9.0 respectively (p = 0.035). The SNR of the lactate signal (black) in animals anesthetized by isoflurane only was calculated to be 8.7 ± 2.6 (mean ± SD) and was calculated with LB =0 from the sum of the 64 spectra as in the sum analysis in Figure 1 in the main text. (**b**) A plot of SNR versus endogenous HDO signal indicates a linear relationship between the HDO signal and the observed SNR. Note that the points designated by the dashed circle were excluded from the linear fit. This is in line with previous report that in preclinical MR measurements the main contributor to the noise is the RF instrumentation and we can assume that the noise level is similar in all animals investigated in this study. Individual values from animals anesthetized by MED-ISO and ISO anesthesia are in red and green respectively.
